# Tumor Microenvironment and Genes Affecting the Prognosis of Temozolomide-Treated Glioblastoma

**DOI:** 10.3390/jpm13020188

**Published:** 2023-01-20

**Authors:** Yena Jang, Wooyong Cheong, Gyurin Park, Yeongmin Kim, Junbeom Ha, Sangzin Ahn

**Affiliations:** 1 Inje University College of Medicine, Busan 47392, Republic of Korea; 2 Department of Pharmacology and Pharmacogenomics Research Center, Inje University College of Medicine, Busan 47392, Republic of Korea

**Keywords:** glioblastoma, temozolomide, tumor microenvironment, differentially expressed genes, precision medicine, pharmacogenetics, transcriptome

## Abstract

Glioblastoma (GBM) is the most frequent primary brain tumor in adults and has a poor prognosis due to its resistance to Temozolomide (TMZ). However, there is limited research regarding the tumor microenvironment and genes related to the prognosis of TMZ-treated GBM patients. This study aimed to identify putative transcriptomic biomarkers with predictive value in patients with GBM who were treated with TMZ. Publicly available datasets from The Cancer Genome Atlas and Gene Expression Omnibus were analyzed using CIBERSORTx and Weighted Gene Co-expression Network Analysis (WGCNA) to obtain types of highly expressed cell types and gene clusters. Differentially Expressed Genes analysis was performed and was intersected with the WGCNA results to obtain a candidate gene list. Cox proportional-hazard survival analysis was performed to acquire genes related to the prognosis of TMZ-treated GBM patients. Inflammatory microglial cells, dendritic cells, myeloid cells, and glioma stem cells were highly expressed in GBM tissue, and *ACP7*, *EPPK1*, *PCDHA8*, *RHOD*, *DRC1*, *ZIC3*, and *PRLR* were significantly associated with survival. While the listed genes have been previously reported to be related to glioblastoma or other types of cancer, *ACP7* was identified as a novel gene related to the prognosis of GBM. These findings may have potential implications for developing a diagnostic tool to predict GBM resistance and optimize treatment decisions.

## 1. Introduction

Glioma is a tumor originating from neuroglial cells of the brain and spinal cord, and is known for its poor prognosis [1]. Glioma is classified into four malignancy grades based on histological criteria, such as atypia and necrosis. Among them, glioblastoma (GBM) is the most frequent and malignant grade 4 tumor [2]. Surgery is the most effective treatment for GBM, albeit the challenging location and capability to infiltrate into surrounding healthy tissue make it difficult [3]. In addition, remnants must be treated with chemotherapy and radiation therapy post-surgery [4]. For chemotherapy of GBM, the cytotoxic anticancer drug Temozolomide (TMZ) is the drug of choice. Unfortunately, due to the highly heterogeneous and mutation-prone nature of GBM, more than half of patients do not respond to TMZ, limiting the median survival to 12–15 months [5].

TMZ resistance is a significant obstacle that must be tackled for the successful treatment of GBM. Previous studies have shown that glioma stem cells and the MGMT repair system play key roles in TMZ resistance, and that PI3K/AKT, Wnt/b-catenin, and JAK/STAT pathways are also involved [6,7,8]. However, limited research has been conducted on GBM transcriptome data to investigate tumor microenvironments and genes that affect prognosis in GBM patients treated with TMZ [9].

In this study, we aimed to identify putative transcriptomic biomarkers with predictive value in TMZ-treated GBM patients using GBM single-cell RNA (scRNA) sequencing data, as well as clinical information and gene expression data from publicly available databases. To take the tumor microenvironment into account, we used Cell-type Identification By Estimating Relative Subsets Of RNA Transcripts (CIBERSORTx) to identify highly expressed cell types, and employed Weighted Gene Co-expression Network Analysis (WGCNA) to construct gene modules that are highly correlated with these cell types. We also used Differentially Expressed Genes (DEG) analysis on short and long survival groups, and intersected the resulting lists to obtain a candidate gene list. Finally, survival analysis was performed to analyze these genes as biomarkers.

## 2. Materials and Methods

### 2.1. scRNA Sequencing Data: GEO Dataset

Glioblastoma single-cell RNA (scRNA) sequencing dataset GSE162631 was downloaded from the Gene Expression Omnibus (GEO) database. This dataset included sequences of tumor core and peripheral tissue from 4 GBM patients, which were processed using magnetic-activated cell sorting to separate endothelial cell marker CD31-positive cells.

### 2.2. Bulk RNA Sequencing Data and Clinical Data: TCGA Dataset

Public clinical data and gene expression data of patients meeting the criteria were downloaded from The Cancer Genome Atlas (TGCA) database using R packages “TCGAbiolinks” and “SummarizedExperiment” [10,11,12,13]. Data from patients who have undergone only TMZ chemotherapy were collected. A total of 35 available samples in the TCGA-GBM cohort were selected for further analysis. Since the survival information was right-censored data, preprocessing was performed under the assumption of exponential distribution using the mean residual lives to estimate the survival time of censored data before use, except for the final survival analysis. The patients were divided into 3 survival groups: “short survival group” with survival of fewer than 180 days, “medium survival group” with survival from 180 to 730 days, and “long survival group” with survival of more than 730 days based on two points of discontinuation (Figure S1, Table S1a). The short and long survival groups were compared in further analyses.

### 2.3. scRNA Sequencing Data Analysis

The R package “Seraut” was used to analyze 8 scRNA sequence data from the GSE162631 dataset [14]. The original data contained 120,218 cells. The percentages of mitochondria and ribosomal RNA were calculated using the “PercentageFeatureSet” function. Cells were filtered in if they had genes greater than 200, a count of RNA per cell greater than 500, and a percentage of mitochondrial read less than 20%. After filtering, a total of 112,359 cells remained. Next, each of the sequencing data were normalized through log normalization, then merged using the “IntegrateData” function.

The Merged data were scaled using the “ScaleData” function, then dimension was reduced using the “RunPCA” function with the first 2000 highly variable genes screened through the “FindVariableFeatures” function. Subsequently, the top 50 principal components were selected to conduct additional dimension reduction using the UMAP method. We used the “FindNeighbors” and “FindClusters” functions with resolution = 1.0, resulting in 30 clusters. Finally, we used the “FindAllMarkers” function with logfc = 0.5, minpct = 0.35 to find the top 10 marker genes of each cluster. Marker genes were screened using the corrected *p*-value under 0.05, then we used cellKb database (https://www.cellkb.com/ (accessed on 19 October 2022)) to identify each cluster by cell type [15].

### 2.4. CIBERSORTx

CIBERSORTx (https://cibersortx.stanford.edu/ (accessed on 19 October 2022)) is a machine learning algorithm developed by Stanford, which accurately estimates the relative proportions of cell subsets in tissue bulk RNA sequencing data based on the input of scRNA sequencing data matrix [16,17,18]. The difference in cell ratio of each sample was visualized using “ggplot2”. Based on the gene signature matrix from the result of CIBERSORTx, cells with high average expression were further studied to learn how intracellular gene expression characteristics affect prognosis.

### 2.5. WGCNA

WGCNA creates a weighted correlation network to identify modules based on gene expression data and finds modules with the highest correlation to the trait data [19]. The “blockwiseModules” function was used with power = 7, minModuleSize = 30, maxBlockSize = 3000, mergeCutHeight = 0.3.

### 2.6. DEG and Functional Pathway Analysis

DEG analysis was performed between the short and long survival groups [20,21,22]. Next, we overlapped the gene modules that had the highest correlation with each selected cell from CIBERSORTx with genes upregulated in either short or long survival groups from DEG analysis. Functional pathway analysis was performed using the “clusterProfiler” package [23,24].

### 2.7. Survival Analysis

Survival analysis was performed using the overlapping genes from the previous step to select significant genes. Analysis and visualization were performed using “survival”, “survminer” packages [25]. First, univariate analysis was performed to select genes with *p*-value < 0.05, and then analyzed with Cox proportional-Hazard survival analysis using the stepwise variable selection method, resulting in a regression equation and verification of the significance of the variables. “My.stepwise.coxph” function from the “My.stepwise” package was used for variable selection, with sle = 0.05, sls = 0.05.

## 3. Results

### 3.1. Highly Expressed Cell Types and Related Gene Modules

**Figure 1:**
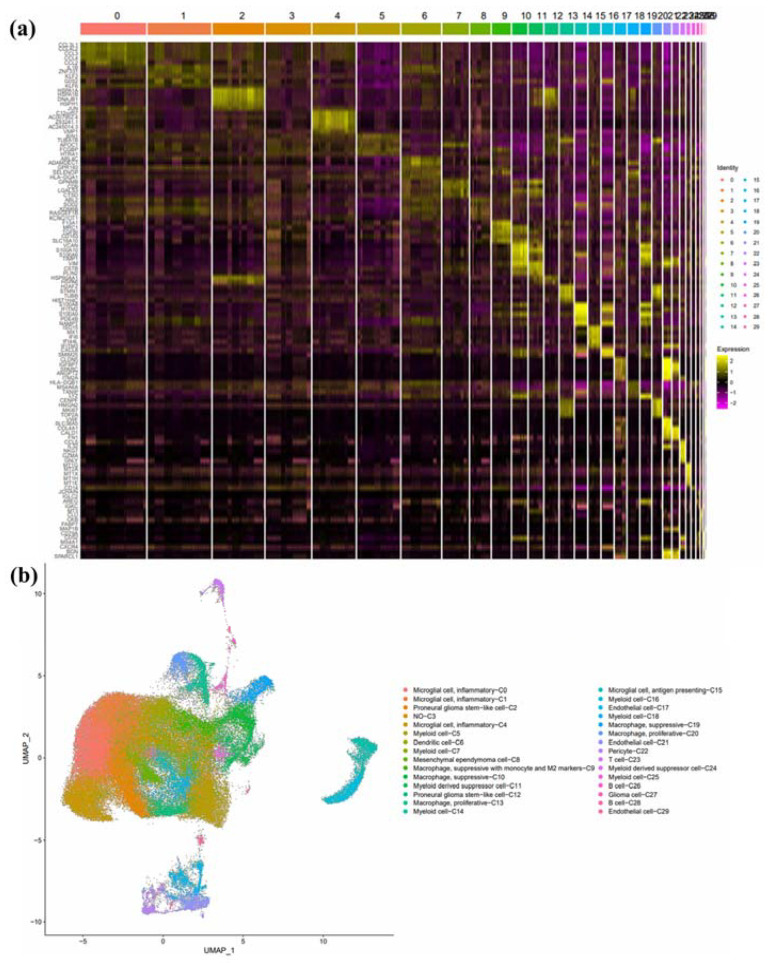
Cell clusters acquired from single-cell RNA sequencing analysis. The 30 clusters resulting from single-cell RNA sequencing analysis were visualized by (**a**) heatmap and (**b**) UMAP.

Thirty cell clusters were acquired from scRNA sequencing analysis and annotated with cell types (Figure 1). The top five marker genes in each cluster are available in Table S1b. By applying CIBERSORTx on the scRNA sequencing GEO dataset and TCGA-GBM RNA expression of 32 GBM samples, the relative proportions of cell types were obtained. There were seven types of cells with a prevalence higher than 5%, and these cell types were selected for further analysis of their gene expression patterns. The selected cell types were Dendritic cell_C6, Inflammatory microglial cell_C0, Inflammatory microglial cell_C1, Inflammatory microglial cell_C4, Myeloid cell_C5, Myeloid cell_C7, and Proneural glioma stem-like cell_C2, as shown in Table S1c.

**Figure 2:**
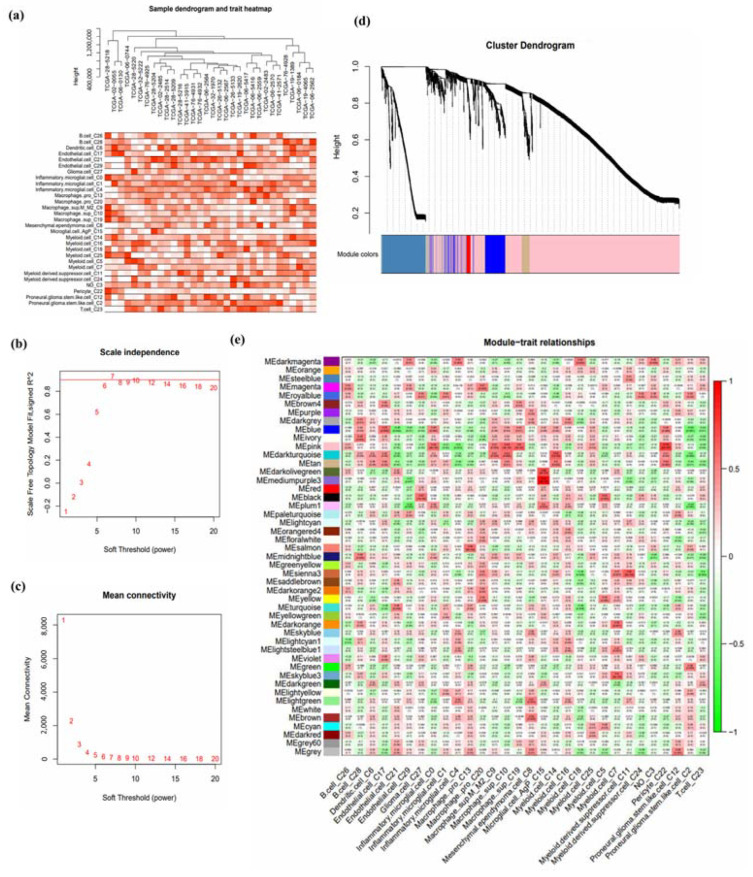
Weighted Gene Co-expression Network Analysis. WGCNA was conducted to investigate the correlation between the fraction of the chosen cell groups and RNA expression from 35 TMZ-treated patients. (**a**) The correlation was demonstrated in the clustering dendrogram. (**b**,**c**) Power = 7 was chosen as the appropriate soft power value. A total of 47 modules were constructed and visualized as a (**d**) cluster dendrogram and (**e**) correlation heatmap.

WGCNA was conducted to explore the correlation between the fraction of the chosen cell groups and RNA expression from 35 TMZ-treated patients. Data from 32 samples were used, with the exclusion of three outliers. The correlation between samples and traits is demonstrated in the clustering dendrogram (Figure 2a). Power = 7 was chosen as the appropriate soft power value (Figure 2b,c). A total of 47 modules were constructed and visualized as a cluster dendrogram and correlation heatmap (Figure 2d,e). Next, modules with the highest correlation and lowest *p*-value with the top seven highly expressed cells were selected (Table 1). The highest correlated gene modules were pink, royalblue, darkmagenta, darkturquoise, black, skyblue3, and green.

### 3.2. Survival-Related Genes and Functions

To explore genes related to survival in TMZ-treated GBM patients, we conducted DEG analysis between the two groups of different survival prognoses. The cut-off criterion was set as |log2 fold change| > 1 and false discovery rate (FDR) < 0.05. A list of 752 differentially expressed genes (high DEG: 151 genes upregulated in the long survival group and low DEG: 601 genes upregulated in the short survival group) was obtained.

**Figure 3:**
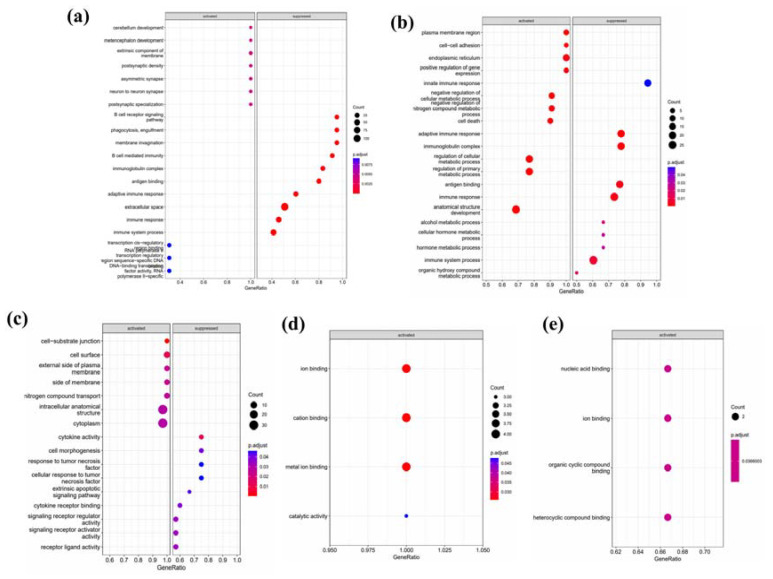
Functional pathway analysis of differentially expressed genes. (**a**) Biological and functional pathways of the genes upregulated in the short survival group were analyzed. Genes mediating neural development were upregulated, and genes mediating immune response were downregulated. Functional pathway analysis of the genes in (**b**) group 1, (**c**) group 2, (**d**) group 4, and (**e**) group 5 was also performed.

Functional pathway analysis revealed alterations in biological pathways based on the differentially expressed genes. Pathways related to neural development were upregulated, and those mediating immune responses were downregulated (Figure 3a). To classify the difference in pathways according to the cell types, genes from the DEG results that overlap with the gene modules identified by WGCNA were organized as shown in Table 1. This process reduced the number of candidate genes from 752 to 264.

The genes were grouped according to cell type and good or bad DEG, resulting in six arbitrary subgroups, and then subjected to functional pathway analysis. In group 1, genes related to the plasma membrane region, cell-to-cell adhesion, upregulation of gene expression, suppression of cellular metabolism, nitrogen compound metabolism, cell death, and anatomical structure development were upregulated, and genes regarding innate immune responses, antigen binding, and hormone metabolic process were downregulated (Figure 3b). In group 2, genes related to the cell–substrate junction, cell surface, plasma external membrane composition, nitrogen compound transport, intracellular anatomical structures, and cytoplasm were upregulated, and genes regarding cytokine activity, cell morphogenesis, response to RNF, extrinsic apoptotic pathway, cytokine receptor binding, signaling receptor activity were downregulated (Figure 3c).

In groups 3 and 4, since the analysis using a threshold of significance level 0.05 did not yield any significant results, a threshold of 0.10 was used. In group 4, genes related to cation binding and catalytic activity were upregulated (Figure 3d). In group 5, genes related to nucleic acid binding, ion binding, organic cyclic compound, and heterocyclic compound binding were upregulated (Figure 3e). In group 6, no significant pathway was returned due to the low number of genes.

### 3.3. Survival Analysis Results

With the 264 candidate genes that were both present in DEG results and gene modules of the most prevalent cell types, survival analysis was performed to determine the genes that significantly contribute to survival in TMZ-treated patients. Firstly, univariate Cox regression was performed to select genes with *p*-values less than 0.05. Among the 90 selected genes, 66 genes that have a reported “external_gene_name” and have published literature related to human disease in Pubmed, the Cochrane library, or EMBASE were selected. Next, multivariate cox analysis utilizing stepwise variable selection was performed. The significance of the model and genes was evaluated by the Wald test and the *p*-value of each gene. Finally, the Cox proportional-hazard assumption was tested using a time-dependent Cox proportional-hazard model, including significant covariates, and confirmed that the model satisfies the assumption.

The final cox proportional-hazard model revealed that *ACP7*, *EPPK1*, *PCDHA8*, *RHOD*, *DRC1*, *ZIC3*, and *PRLR* were statistically significant. Based on the significant genes in univariable analysis, *ACP7*, *EPPK1*, *PCDHA8*, *RHOD*, *DRC1*, and *PRLR* were significantly associated with a short survival, while *ZIC3* was associated with a long survival. Based on the significant genes of the final model, *ACP7*, *EPPK1*, *PCDHA8*, *RHOD*, and *DRC1* were significantly associated with short survival, while *ZIC3* and *PRLR* were associated with long survival when compared with the genes with positive coefficients. The hazard ratios are described in Table 2.

## 4. Discussion

In this study, a deconvolution method was used to evaluate the cell type prevalence in GBM tissue. This information was then utilized to identify genes associated with prognosis in patients with GBM who were treated with TMZ. Integrating these results with DEG analysis between long and short survival groups narrowed down the candidate genes, and the final cox model revealed *ACP7*, *EPPK1*, *PCDHA8*, *RHOD*, *DRC1*, *ZIC3*, and *PRLR* as putative biomarkers. These findings are congruent with the previous literature, as *ZIC3*, *PCDHA8*, *PRLR*, and *DRC1* have been reported to affect survival in GBM or types of gliomas, and *EPPK1* and *RHOD* have been previously connected to tumorigenesis or tumor invasion in other types of cancers. It is noteworthy that, to the authors’ best knowledge, this study is the first to demonstrate that *ACP7* has an influence on the survival of GBM patients.

In particular, *ZIC3* was associated with longer survival, which is consistent with previous studies that have shown that *ZIC3* was downregulated in malignant high-grade glioma [26]. *PCDHA8*, a member of the protocadherin alpha family, was associated with shorter survival. It is known to participate in neural cadherin-like adhesion, serving a key role in brain cell connection [27], and previous studies have reported that *PCDHA8* is hypermethylated in gliomas [28]. Another study reported that the PCDH-gamma-A11 gene is hypermethylated in astrocytoma and inactivates cell-to-cell contact in the brain, causing astrocytoma invasion [27]. The relationship between *PCDHA8* promoter methylation and its expression, and its effect on GBM survival, needs to be evaluated through further studies. As for *PRLR*, prolactin (PRL) has been traditionally associated with lactation and fertility, but recently it has been reported to promote tumor cell proliferation, angiogenesis, and chemoresistance [29]. *PRLR* expression alone was associated with shorter survival in univariate analysis, but was associated with a longer survival in the final Cox proportional-hazard model. A previous study reported that *PRLR* activation increased proliferation, chemoresistance, and matrix metalloproteinase activity in GBM cells [30]. However, as PRL increases when the dopamine pathway is inhibited, infiltration to the pituitary stalk region may be the underlying cause of association with poor prognosis. Present results showed that *DRC1*, dynein regulatory complex subunit 1, expression is associated with short survival, which was partially in line with a previous study reporting that circRNAs derived from *DRC1* were upregulated in ependymomas [31]. Regarding *EPPK1*, epiplakin1, it is generally known to participate in epidermal growth factor signaling and cell proliferation, as well as cytoskeleton reorganization. Our data showed that *EPPK1* expression is associated with short survival, which is partially coherent with previous studies that have shown that *EPPK1* expression activates cell proliferation in cervical cancer and esophageal squamous cell carcinoma [32,33]. As for *RHOD*, ras homolog family member D, our study showed that *RHOD* expression is associated with short survival, which is partially in accord with a previous study that reported that the *RHOD* promotor was differentially methylated between pituitary adenoma and normal tissue [34]. Lastly, *ACP7*, acid phosphatase 7, has not been previously reported to play a role in tumorigenesis or TMZ resistance, but according to the Human Protein Atlas, *ACP7* is highly expressed in head and neck cancer and lung cancer [35].

CIBERSORTx results showed that the most abundant cell types in GBM were inflammatory microglial cells, dendritic cells, myeloid cells, and glioma stem cells. Glioma stem cells (GSCs) are pluripotent cells that lead to short survival and relapse of GBM, and are known to cause TMZ resistance through their slow mitosis [8]. Microglial cells are known to function as antigen-presenting cells (APCs), recognizing tumor cells and inducing cytotoxic T cells to kill tumor cells [36,37]. Recent studies focused on microglial cell polarization to convert microglial cells to an antitumor phenotype suggest its therapeutic potential [38]. Dendritic cells have been suggested to play roles in the inflammation of the brain [39]. The marker genes of dendritic cells were *ARL4C*, *HLA-DQA1*, *IL1B*, *HLA-DQB1*, and *CD70*, where HLA-DQ is an MHC class 2 surface receptor of APCs, IL1B is a lymphocyte activating cytokine, and ARL4C is a GTPase that regulates cell migration [40]. CD70 is known to be activated in mature dendritic cells and to play a key role in recurrent GBM cell aggressiveness and maintenance [41]. Myeloid cells are known to contribute to the GBM microenvironment by regulating immune and therapeutic responses [42]. Genes such as *DRC1*, which are known to be relevant to myeloid cells and to be upregulated in ependymomas, are worth further investigation for their prognostic value [31].

The limitations of this study include the lack of comparison between patients treated and not treated with TMZ, which would have strengthened the evidence for the specificity of the identified biomarkers. As TMZ is the first-line drug for GBM, most transcriptomic datasets are derived from the tissue of patients who have already undergone TMZ treatment. Additionally, the sample size of tissue analyzed in this study was small, and there was no functional validation of the identified genes. These shortcomings highlight the need for future research, which should include larger sample sizes, as well as functional validation of the biomarkers through experimental studies. Additionally, a comparison between groups of treated and untreated patients would provide further evidence for the biomarkers identified and their potential as a prognostic indicator.

## 5. Conclusions

In conclusion, this study identified that inflammatory microglial cells, dendritic cells, myeloid cells, and glioma stem cells are highly expressed in GBM tissue. Additionally, through the use of a deconvolution method and DEG analysis, this study also identified a gene signature consisting of *ACP7*, *EPPK1*, *PCDHA8*, *RHOD*, *DRC1*, *ZIC3*, and *PRLR* that are associated with poor prognosis in patients with GBM who were treated with TMZ. These findings may have potential implications for developing a diagnostic tool to predict TMZ resistance in GBM patients and optimize treatment decisions. However, further research is necessary to confirm these findings and to explore the underlying mechanisms that these cell types and genes play in GBM progression.

## Figures and Tables

**Table 1:** WGCNA and DEG results on top seven highly expressed cells.

Cell Type	Module	Correlation	*p*-Value	Total Genes ^1^	High DEG ^2^	Group	Low DEG ^3^	Group
Inflammatory microglial cell_C0	Pink	0.5595	0.00087135	1996	0	Group 5	156	Group 1
Inflammatory microglial cell_C1	Royalblue	0.4386	0.01203565	1190	10	Group 5	1	Group 1
Inflammatory microglial cell_C4	Darkmagenta	0.4542	0.00902103	223	2	Group 5	0	Group 1
Dendritic cell_C6	Darkturquoise	0.4016	0.02271183	986	0		70	Group 2
Myeloid cell_C5	Black	0.5303	0.00179763	2063	1	Group 6	8	Group 3
Myeloid cell_C7	Skyblue3	0.5974	0.00030639	511	0	Group 6	0	Group 3
Proneural glioma stem like cell_C2	Green	0.4565	0.00864020	2767	0		16	Group 4

^1^ Total number of genes in the gene module. ^2^ Number of genes that overlap with the differentially expressed genes in high survival group. ^3^ Number of genes that overlap with the differentially expressed genes in low survival group.

**Table 2:** Cox proportional-hazard analysis results of 66 candidate genes.

Group	External_Gene_Name	Entrezgene_Description	Hazard Ratio	Lower 0.95 of CI ^1^	Upper 0.95 of CI ^1^	*p*-Value ^2^	Hazard Ratio of Univariate Analysis
Group 5	ZIC3	Zic family member 3	0.9480547	0.9203	0.9766	0.000428 ***	0.9743351
Group 4	ACP7	acid phosphatase 7, tartrate resistant (putative)	1.0906662	1.0406	1.1432	0.000295 ***	1.0273678
Group 2	EPPK1	epiplakin 1	1.0419592	1.0196	1.0648	0.000209 ***	1.018163
Group 4	PCDHA8	protocadherin alpha 8	1.0293742	1.0122	1.0468	0.000748 ***	1.0097472
Group 2	PRLR	prolactin receptor	0.9549296	0.9316	0.9789	0.000261 ***	1.0077297
Group 2	RHOD	ras homolog family member D	1.0079418	1.0039	1.012	9.96 × 10^−5^ ***	1.0017014
Group 3	DRC1	dynein regulatory complex subunit 1	1.0020724	1.0006	1.0036	0.007405 **	1.001401

^1^ Confidence interval. ^2^ ** *p* < 0.01, *** *p* < 0.001.

## Data Availability

The data that support the findings of this study are available from the Cancer Genome Atlas (TCGA, http://cancergenome.nih.gov/ (accessed on 19 October 2022)) and Gene Expression Omnibus (GEO, https://www.ncbi.nlm.nih.gov/geo/, accession number GSE162631 (accessed on 19 October 2022)).

## Supplementary Materials

The following supporting information can be downloaded at https://www.mdpi.com/article/10.3390/jpm13020188/s1. Figure S1: survival information; Table S1a: demographic information of good, middle, bad survival groups of 35 patients; Table S1b: top five highly expressed genes of each cell type identified by CIBERSORTx; Table S1c: CIBERSORTx results; and Table S1d: literature review for significant genes of subgroups.
