# Supplementary material for: Tumor Microenvironment and Genes Affecting the Prognosis of Temozolomide-Treated Glioblastoma
Source: J Pers Med. 2023 Jan 20;13(2):188. doi: 10.3390/jpm13020188 (PMC9966340; doi:10.3390/jpm13020188)
Supplement: Supplementary file 1 [file jpm-13-00188-s001.zip › Supplementary_Figure_S1.pdf]

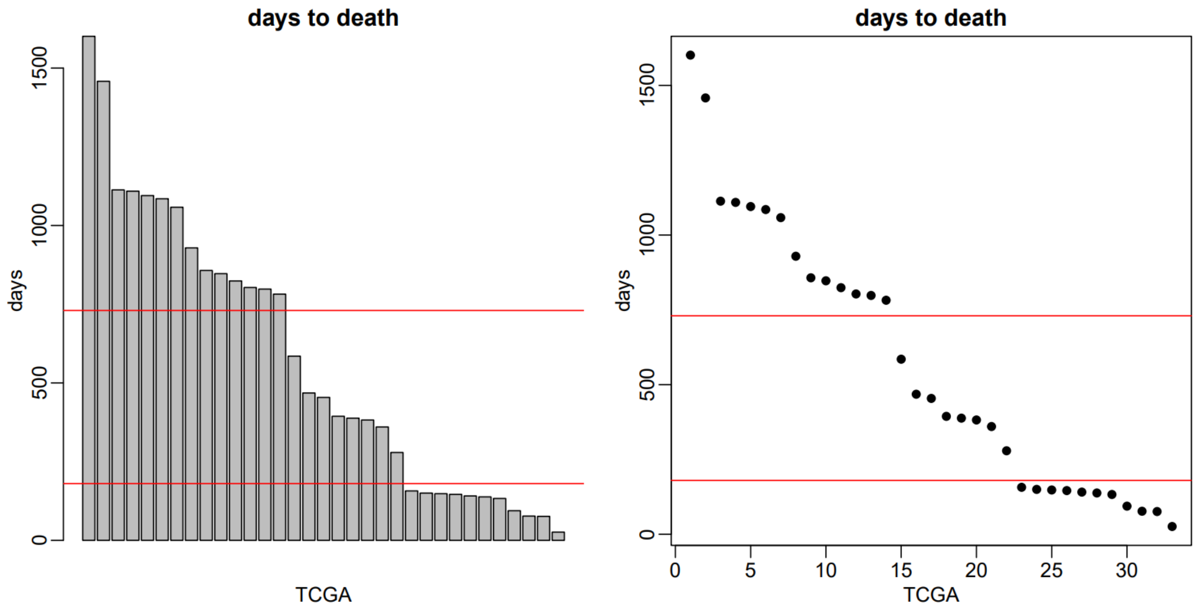

Figure S1. Since the survival information was randomly censored data, preprocessing was performed under the assumption of exponential distribution using the mean residual lives to estimate the survival time of censored data before use, except for the survival analysis. The patients were divided to 3 survival groups, "short survival group" with survival of less than 180 days, "medium survival group" with survival from 180 to 730 days, and "long survival group" with survival of more than 730 days based on two points of discontinuation.

Title: Prognostic Gene Signature and Tumor Microenvironment for Temozolomide Treatment of Glioblastoma Based on Single-Cell Transcriptomes

Journal name: Clinical and Translational Oncology

Authors: Yena Jang[1*], Wooyong Cheong[1*], Gyurin Park[1], Yeongmin Kim[1], Junbeom Ha[1], Sangzin Ahn[2]

Affiliations:

[1]Inje University College of Medicine, Busan, Republic of Korea

[2]Department of Pharmacology and Pharmacogenomics Research Center, Inje University College of Medicine, Busan, Republic of Korea

[*]Yena Jang and Wooyong Cheong contributed equally to this work

Corresponding author: Correspondence to Sangzin Ahn (sangzinahn@gmail.com)
